# Circulating mRNA Profiling in Esophageal Squamous Cell Carcinoma Identifies FAM84B As A Biomarker In Predicting Pathological Response to Neoadjuvant Chemoradiation

**DOI:** 10.1038/srep10291

**Published:** 2015-05-18

**Authors:** Feng-Ming Hsu, Jason Chia-Hsien Cheng, Yih-Leong Chang, Jang-Ming Lee, Albert C. Koong, Eric Y. Chuang

**Affiliations:** 1 Graduate Institute of Biomedical Electronics and Bioinformatics, National Taiwan University, Taipei, Taiwan; 2 Graduate Institute of Oncology, National Taiwan University, Taipei, Taiwan; 3 Cancer Research Center, National Taiwan University, Taipei, Taiwan; 4 Bioinformatics and Biostatistics Core, Center of Genomic Medicine, National Taiwan University, Taipei, Taiwan; 5 Department of Oncology, National Taiwan University Hospital; 6 Department of Pathology,National Taiwan University Hospital; 7 Department of Surgery, National Taiwan University Hospital; 8 Department of Radiation Oncology, Stanford University School of Medicine, Stanford, California, United States

## Abstract

Esophageal cancer patients with pathological complete response (pCR) to neoadjuvant chemoradiation (CRT) have favorable outcomes. Currently, there was no reliable biomarker predicting the response to CRT. Perioperative circulating mRNA may be associated with prognosis, but its application for predicting treatment response is unclear. We prospectively assessed the value of circulating messenger RNA (mRNA) profiling in predicting pCR for esophageal squamous cell carcinoma (ESCC). Patients with ESCC completing CRT followed by surgery were enrolled for analysis. Venous peripheral blood was obtained before and after CRT, and total RNA was extracted for hybridization-based whole genome expression analysis and quantitative RT-PCR. We found circulating expression profiling was significantly altered after CRT. Altered FAM84B expression was significantly predictive of pCR. The decrease of serum FAM84B protein level after CRT was also associated with pCR. Immunohistochemistry and western blot confirmed that FAM84B protein was overexpressed in the majority of patients and ESCC cell lines. Furthermore, knockdown of FAM84B delayed tumor growth in ectopic xenografts. We demonstrated the decreased of circulating FAM84B mRNA and protein after neoadjuvant CRT may predict pCR, and FAM84B protein is overexpressed in ESCC. The potential of FAM84B as a novel predictive biomarker, and its biological functions deserve further investigation.

Compared to surgery alone, neoadjuvant chemoradiation (CRT) followed by curative surgery improves the absolute 2-year survival rate of both squamous cell carcinoma (SCC) and adenocarcinoma of the esophagus^1^. Among patients underwent combined modality therapy, pathological complete response (pCR) to neoadjuvant CRT is the most important prognostic factor associated with a better overall survival^2^. Currently, no predictors of response to preoperative treatment based on standard pathological assessment are reliable^3^. Although the preliminary results of studies using high-throughput technologies to identify novel molecular biomarkers or signatures are promising and encouraging, further investigation and validation are needed^4^,^5^,^6^,^7^,^8^. However, the underlying biological mechanisms of identified biomarkers in these studies remained unclear.

On average, only 20–30% of patients achieve pCR after neoadjuvant treatment^2^,^9^,^10^. It is therefore important to identify factors predictive of treatment response so that therapy can be personalized to maximize therapeutic ratio and more effective regimens can be developed in the future.

Specific circulating messenger RNAs (mRNAs) were found to predict the postoperative prognosis and histopathological response to neoadjuvant CRT in esophageal cancer^11^,^12^,^13^. Till now, little data have been collected on the correlation of whole blood transcriptomes with treatment response. Oshita *et al.* used genome-wide cDNA microarrays to identify certain genes in peripheral blood cells predictive of the benefits of chemotherapy in patients with non-small cell lung cancer^14^, suggesting circulating mRNAs might be useful biomarkers in predicting treatment response.

In previous study, we successfully identify two germline single nucleotide polymorphisms (SNPs) predictive of pCR to neoadjuvant CRT in esophageal SCC (ESCC) from peripheral blood^7^. Herein, we report a prospective evaluation on the alteration of circulating mRNA profiles before and after neoadjuvant treatment, its role in predicting pathological response to neoadjuvant CRT, and the biological source of identified novel biomarker.

## Results

### Clinical outcome of studying patients

The characteristics of the 37 patients are shown in Table 1. Samples of 21 patients were tested by both microarray and RT-PCR, while those of 16 patients were tested by RT-PCR alone. A pCR after neoadjuvant CRT was achieved in 16 patients (43%).

With a median follow-up of 38 months, the median overall survival and progression-free survival were 42 months and 34 months, respectively. In univariate analyses, non-pCR and pathological lymph node metastasis predicted poor overall survival (*p* = 0.009 and *p* <0.001, respectively) and progression-free survival (*p* = 0.009 and *p* = 0.002, respectively).

### Circulating mRNA profiles differ before and after CRT

**Figure 1:**
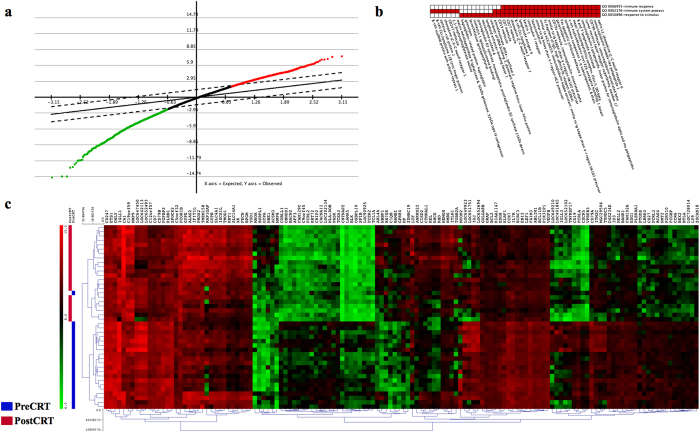
The circulating mRNA profiles were significantly altered between before and after preoperative chemoradiation (CRT). (**a**) Significance Analysis of Microarray (SAM) plot. (**b**) The gene ontology term enrichment analysis was performed by the Database for Annotation, Visualization, and Integrated Discovery. Red and white colors denote the positively and negatively reported corresponding gene-annotation association, respectively. (**c**) Supervised hierarchical cluster analysis identified 136 mRNAs with expression that differed significantly between before and after CRT. Green and red colors denote down- and up-regulated genes, respectively.

Using an FDR of 0.5% and a fold-change more than 2, Significance Analysis of Microarrays (SAM) identified 136 genes differentially expressed between before and after CRT (Fig. 1a). Among the differentially expressed genes, 46 genes were up regulated and 90 genes were down regulated. The gene ontology (GO) term enrichment and functional annotation analysis by Database for Annotation, Visualization, and Integrated Discovery (DAVID) identified 5 significant clusters with the one-tail Fisher exact probability value of 0.025 in the annotation category of all GO biological progress terms. The enriched clusters and related gene groups were summarized in Supplementary Table e1. The annotation cluster one consists of 41 genes and the gene-annotation association map is shown in Fig. 1b. The immune system process and immune response were the two most significant functions altered by CRT (Bonferroni adjusted *p* value <0.001). The supervised hierarchical clustering analysis revealed nearly perfect segregation of the pre-CRT from the post-CRT samples and the resulting heat map is shown in Fig. 1c.

### Expressed circulating mRNAs differ between complete responder and non-complete responder

By using stringent statistical methods, there was no significant difference in the expression profiles between the CR group and non-CR group. Since the neoadjuvant CRT significantly altered the gene expression signature of peripheral blood cells, we hypothesized that changes in expression of circulating mRNA with neoadjuvant CRT may be predictive for pathological response. BAM identified ten candidate mRNAs (AFTPH, C10ORF76, CCNL1, FAM13A1, FAM84B, HIST1H4H, HIST2H4A, IFI27, KCNRG, and SEPT4) for further evaluation (Table 2). A second analysis showed that AFTPH (aftiphilin), CCNL1 (Cyclin-L1), FAM13A1 (family with sequence similarity 13, member A1), FAM84B (family with sequence similarity 84, member B), HIST2H4A (histone cluster 2, H4a), and SEPT4 (septin 4) were truly differentially expressed between patients with pCR and non-pCR.

**Figure 2:**
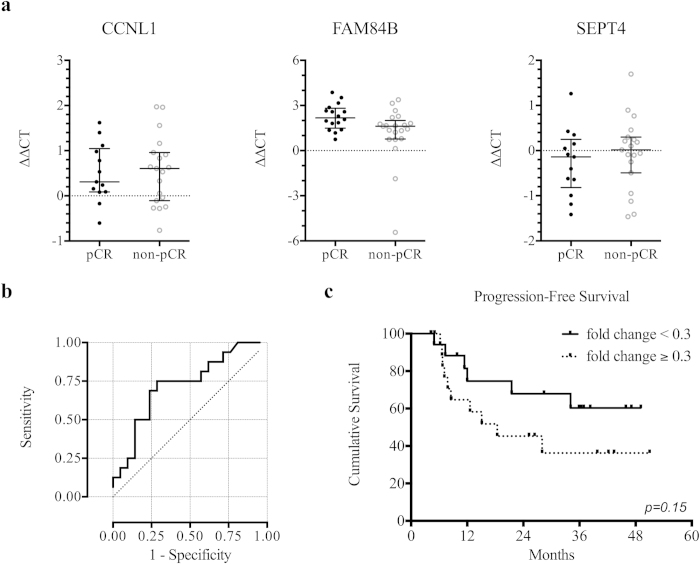
Circulating mRNA in predicting response to and outcome of preoperative chemoradiation. (**a**) The change in circulating levels of CCNL1, FAM84B, and SEPT4 mRNAs between pathological complete responders (pCR) and non-complete responders (non-pCR) measured by quantitative reverse-transcriptase polymerase chain reaction. Data shown as a scatter plot and the intersecting line shows the median value with the interquartile range. (**b**) Receiver-operating characteristic curve shows the performance of fold-change in FAM84B mRNA expression in predicting the pathological complete response, with the area under curve being 0.73. (**c**) Kaplan-Meier curves of the disease-free survival stratified by fold-change in FAM84B mRNA expression using a cutoff value of 0.3 (*p* = 0.15).

The three most differentially expressed circulating mRNAs (CCNL1, FAM84B, and SEPT4) were assayed by quantitative RT-PCR. As shown in Fig. 2a, the change in the expression of FAM84B (*p* = 0.02) but not CCNL1 (*p* = 0.98) or SEPT4 (*p* = 0.41) was significantly different between the pCR and non-pCR groups.

### FAM84B mRNA as a novel biomarker for ESCC

The fold change (2^-∆∆CT^) of circulating FAM84B mRNA obtained by qRT-PCR was used in ROC curve analysis to evaluate its predictive ability. The median value of fold change was 0.3 (ranged 0.1 to 43.1). Figure 2b shows that the area under the two-class ROC curve was 0.73 (*p* = 0.02). With a cutoff value of 0.3, the sensitivity, specificity, positive predictive value, and negative predictive value were 75%, 67%, 63%, and 78%, respectively. Patients with fold change <0.3 had a significantly higher chance to achieve pCR (*p* = 0.01). In survival analysis, patients with fold change <0.3 had a trend toward longer progression-free survival (median not reached versus 18 months, *p* =0.15; Fig. 2c).

### Serum FAM84B protein level change is associated with treatment response

**Figure 3:**
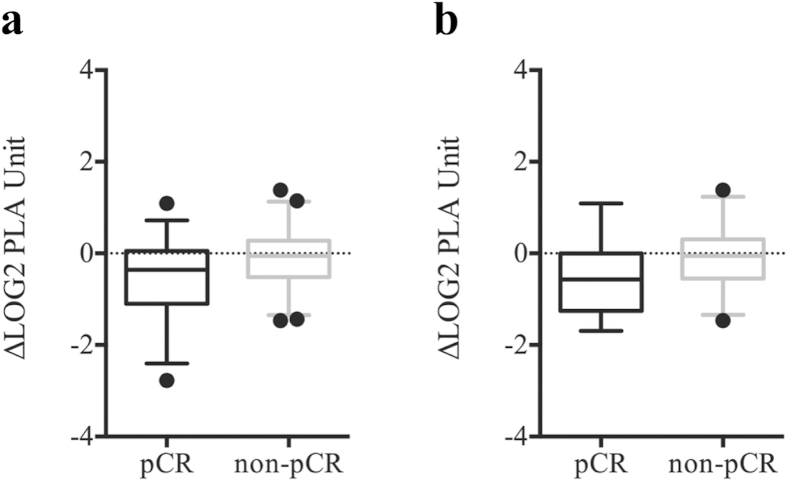
Box-and-Whisker plots at the 5th and 95th percentiles of FAM84B serum protein level quantified by proximity ligation assay. (**a**) The changes after chemoradiation between pathological complete responders (pCR) and non-complete responders (non-pCR) (*p* = 0.02). (**b**) The changes between pCR and non-pCR groups for an independent validation cohort (*p* = 0.06).

To verify that change in circulating FAM84B mRNA is predictive of treatment response, we performed multiplex PLA of serum samples from 79 patients receiving combined modality therapy^15^. Overall, the changes in serum FAM84B protein level showed a trend toward association with pathological response (*p* = 0.08). After removal of two outliers, decrease in serum FAM84B level after CRT was greater in the pCR group than non-pCR group (*p* = 0.02; Fig. 3a). Among the 46 patients in an independent validation cohort, the pCR group (14 patients) had a strong trend toward greater reduction of serum FAM84B protein (*p* = 0.06; Fig. 3b).

### Overexpression of FAM84B in tissue samples of ESCC

**Figure 4:**
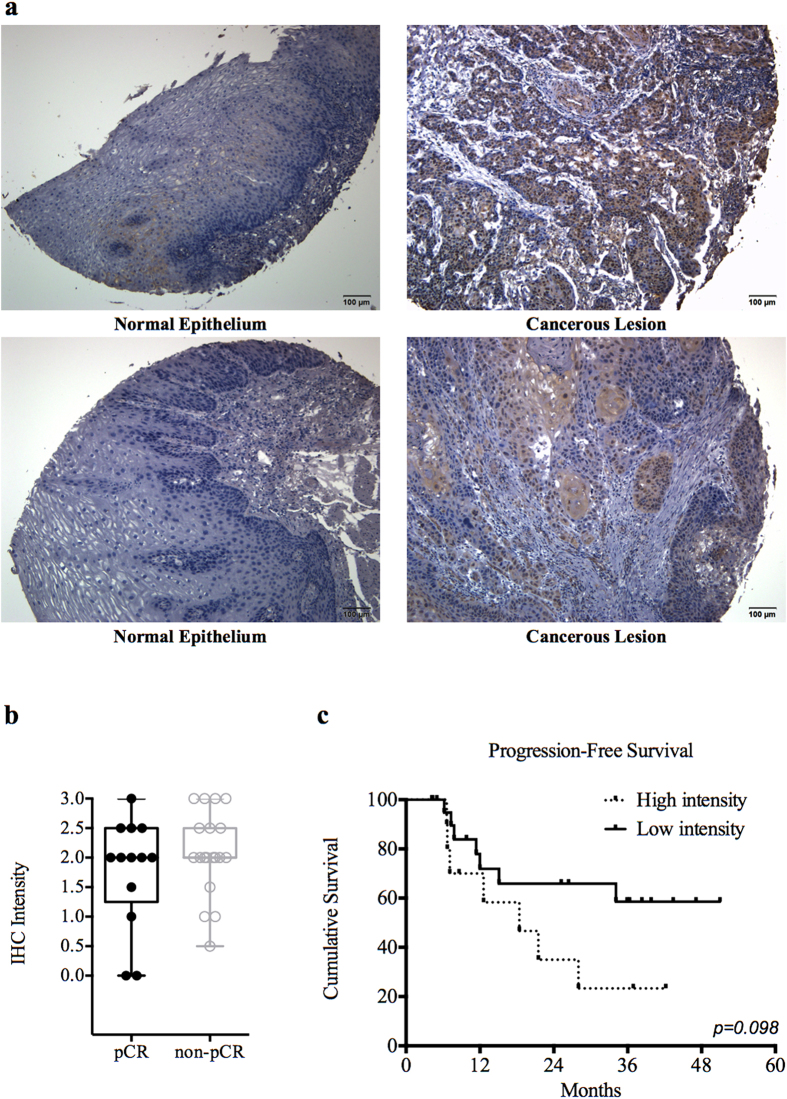
FAM84B was overexpressed in esophageal squamous cell carcinoma (ESCC) tumor biopsies. (**a**) The immunohistochemistry analysis of FAM84B from patients with paired specimens of the cancerous lesion and normal epithelium of the esophagus. (**b**) Box-and-Whisker plot of FAM84B staining intensities between pathological complete responders (pCR) and non-complete responders (non-pCR) (*p* = 0.99). (**c**) Kaplan-Meier curves of the disease-free survival stratified by FAM84B staining intensities (*p* = 0.098).

Thirty-two patients had pretreatment tissue sample available for immunohistochemistry analysis, and 26 samples (81%) showed ≥ moderately staining intensity for FAM84B (Table 1). Seventeen patients had post-esophagectomy residual tumors available for immunohistochemistry analysis, and 16 samples (94%) showed ≥ moderately staining intensity for FAM84B (Table 1). The FAM84B protein was highly expressed in cancerous tissue while the paired normal esophageal epithelium was negative for IHC staining (Fig. 4a). The distributions of FAM84B staining intensities on pretreatment tumor biopsies were not different between pCR and non-pCR groups (*p* = 0.99; Fig. 4b). Interestingly, patients with high intensity on pretreatment tumor biopsies had non-significant worse progression-free survival than those with low intensity (median 18 months versus not reached, *p* = 0.098; Fig. 4c)

### Overexpression of FAM84B in ESCC cell lines

**Figure 5:**
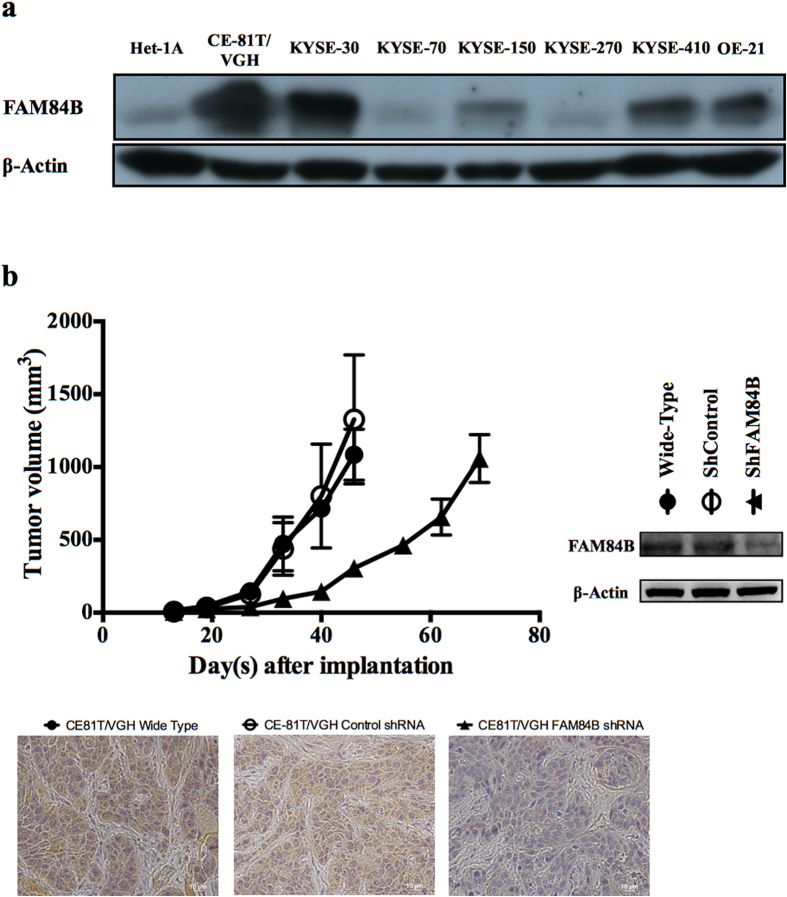
FAM84B was overexpressed in esophageal squamous cell carcinoma (ESCC) cell lines and associated with tumor progression. (**a**) The cropped Western blot of FAM84B protein from lysates of the esophageal non-neoplastic squamous epithelial cell line (Het-1A) and ESCC cell lines (CE46T/VGH & CE146T/VGH are not shown). (**b**) Growth curves of ESCC CE81T/VGH xenograft tumors. Mean volume ±standard deviation (n = 5 per group) are plotted as a function of time since injection. Cropped immunoblots of CE81T/VGH cells transfected with shRNA against FAM84B. Immunohistochemistry stains of tumor xenografts confirmed the FAM84B knockout tumor has no FAM84B protein expression. Images were taken at 40X.

Ten human cell lines were evaluated for FAM84B protein expression by Western blotting. The human esophageal non-neoplastic epithelial cell line Het-1A showed negative or weak expression. Expression by the 9 esophageal SCC cell lines was strong in CE-81T/VGH, KYSE-30, KYSE-410, and OE-21, moderate in KYSE-150, and weak or absent in KYSE-70, KYSE-270, CE-48T/VGH (data not shown), and CE-146T/VGH (data not shown; Fig. 5a). Overall, 56% of esophageal SCC cell lines were positive for FAM84B overexpression.

### Knockdown of FAM84B in xenograft shows delay in ectopic tumor growth

The knockdown of FAM84B expression in ESCC cell line CE81T/VGH showed delay in tumor growth with reduced tumor size of shFAM84B xenografts (96 mm^3^,*p* = 0.003) measured 33 days after subcutaneous injection of cells compared with shControl tumors (438 mm^3^, Fig. 5b). No significant difference between shControl and wild-type tumors was observed (*p* = 0.77).

## Discussion

Neoadjuvant CRT followed by surgery is considered as one of the standard treatments for resectable locally advanced esophageal cancer. However, the role of post-CRT esophagectomy is controversial. Randomized trials concluded that CRT plus esophagectomy (compared with CRT alone) improves local control but not overall survival in patients responding to induction treatment^9^,^10^. Since radical esophagectomy is associated with significant morbidity and risk of mortality, patients with good response to CRT may not need additional surgery^10^,^16^. On the other hand, surgery is a valuable option for patients not responding to CRT^17^. Therefore, early identification of CRT responders should help individualize appropriate strategy and thereby maximize therapeutic effect and minimize treatment-related toxicity.

Currently, there are no clinically reliable predictors of treatment response as judged by standard pretreatment pathological assessment or immunohistochemistry analysis^3^. Sequential metabolic imaging using fluorodeoxyglucose-positron emission tomography does not seem to be applicable when radiation is a component of the neoadjuvant regimens^18^. Ajani *et al.* developed the logistic regression model in 322 esophageal cancer patients with 94% of the cohort having the histological type of adenocarcinoma^19^. The area under the ROC curve was 0.7 and their nomograms consisted of five clinical parameters. In current study, the area under the ROC curve was 0.73, which suggests that biomarker alone may perform as good as complex regression model for predicting pCR.

High-throughput technologies, including microarray and mass spectrometry, provide global information to facilitate systematic discovery of novel biomarkers predicting the response to CRT. The results of preliminary studies using mRNA or microRNA (miRNA) expression from tumor biopsies, though sample size is small, are encouraging^4^,^5^,^8^. Luthra *et al.* used a combination of PERP, S100A2, and SPRR3 expression levels to discriminate pCR from less-than-pCR with high sensitivity and specificity^4^. Maher *et al.* used a class prediction model of 5 genes (EPB4IL3, RNPC1, RTKN, STAT5B, and NMES1) to assess response to CRT and reported 95% accuracy in predicting pCR^5^. Ko *et al.* discovered 111 miRNAs with significantly altered expression after preoperative therapy, and 5 miRNAs in pretreatment tumor samples that were significantly differentially expressed between pCR and non-pCR groups^8^. Further investigation and validation of these studies remain warranted. Of note, the underlying biological mechanisms were not reported for those discovered genes. Moreover, the majority of patients in these studies had adenocarcinoma of the esophagus, which is quite different from SCC in pathogenesis, epidemiology, tumor biology, and prognosis. The histological type of SCC was also associated with higher rate of pCR to neoadjuvant CRT^19^,^20^. Therefore, the prediction models developed in these studies might not be applicable to ESCC. Ashida *et al.* analyzed gene expression patterns in pretreatment biopsy specimens from long-term and short-term survivors after definitive CRT for ESCC^21^. The genes involved in the immune response were characteristically up-regulated in the long-term survivors, while genes involved in drug resistance were overexpressed in the short-term survivors. However, these studies lack consensus on what genes predict the outcome of CRT.

Biomarkers in peripheral blood are of interest as the predictors of response because blood collection is minimally invasive. Maher *et al.* studied serum proteomic profiling in esophageal cancer patients and identified pretreatment serum levels of complement C4a and C3a as biomarkers predictive of treatment response^6^. We also analyzed whole blood for germline SNPs and identified two SNPs (rs16863886 and rs4954256) with a high accuracy for predicting CRT response^7^. These findings merit validation in an independent cohort, but the underlying biological mechanisms require further exploration. In addition to the high-throughput screening for circulating biomarkers, studies using expression of selective genes in peripheral blood showed the detection of CEA, SCC antigen, or survivin mRNAs predicted disease recurrences after surgery^11^,^12^. However, whether these circulating mRNAs predict the response to CRT in esophageal cancer is unknown. Brabender *et al.* reported that the level of ERCC1 mRNA expression in peripheral blood is significantly higher in minor responders than major responders to preoperative CRT^13^. Interestingly, this result is compatible and correlated with findings of those studies using biopsy specimens^22^. Patients with tumors showing no ERCC1 expression by IHC or relatively low expression by RT-PCR were more likely to be major responders. Their finding supports the hypothesis that circulating mRNAs are produced by tissues that release or actively transport them into the bloodstream. These circulating biomarkers, though their origins and underlying mechanisms of release are unclear, may carry important information and could become useful predictors of response.

In the present study, the circulating gene expression profile was significantly altered after CRT. The enrichment annotation analysis showed immune-related functions were changed by CRT. Our finding suggests that the immune system might involve in host response to CRT. Furthermore, circulating FAM84B mRNA was identified as a novel biomarker predicting pathological response to neoadjuvant CRT. Patients with greater reduction of FAM84B mRNA expression in peripheral blood after CRT are more likely to achieve pCR. The analysis of serum FAM84B protein level detected by a highly sensitive technique was in accordance with the FAM84B mRNA finding. Beside, FAM84B protein expression was found in more than 80% of pretreatment tumor biopsy specimens and more than 90% of residual tumors after CCRT. In addition, patients with high IHC intensity on pretreatment tumor biopsies might had worse progression-free survival. However, these findings from IHC analysis should be interpreted carefully since tumor heterogeneity can lead to misinterpretation of the tumor genetic identity from single tumor-biopsy samples^23^. Further investigation using surgical samples from esophagectomy is mandatory to determine the prognostic value of FAM84B expression in clinical outcome. *In vivo*, FAM84B was overexpressed in more than 50% of ESCC cell lines but was not expressed in normal esophageal epithelial cells. Most interestingly, our *in vivo* ectopic xenografts showed knockout of FAM84B results in tumor growth delay. Our data suggests that FAM84B could involve important biological functions in ESCC.

FAM84B (family with sequence similarity 84, member B) was first identified as the breast cancer membrane protein 101^24^. Adam *et al.* detected high levels of FAM84B mRNA and protein in breast carcinoma cells. The protein was widespread intracellularly, but particularly concentrated in plasma membrane areas of cell-cell contact. It was found to interact specifically with α1-catenin protein, which was associated with the cancer cell properties of aberrant cell adhesion and invasion^25^. The FAM84B gene is located in chromosome 8q24.21 (with gene for FAM84B being at the centromeric end and the gene for c-MYC at the telomeric end). Genetic variants of this locus have known associations with susceptibility to prostate, ovarian, and colorectal cancer^26^. Huang *et al.* found amplification of 8q24 in ESCC, but found c-MYC protein expression in part of the esophageal cancerous nest in only 4 of 46 cases by IHC analysis^27^. Interestingly, they found increased expression of FAM84B mRNA in 66% of patients with ESCC, and suggested its involvement in the genesis or development of esophageal cancer in southern China. In contrast, van Duin *et al.* found significantly decreased FAM84B in patients with gastroesophageal junction adenocarcinomas^28^. The conflicting reports implied that the pathogenesis of SCC is distinct from that of esophageal adenocarcinoma.

To date, little is known about the function of FAM84B. It has been identified as one of the estrogen receptor α regulatory genes in breast cancer cell line MCF-7^29^. FAM84B gene expression is down-regulated in pancreatic cancer cells treated with the heat shock protein 90 inhibitor, IPI-504, and in BRAF mutant melanoma cells treated with the MEK inhibitor, PD-032590^30^,^31^. In addition, FAM84B gene expression is suppressed in cell line transfected with HIPK2 expression vector, which inhibits cancer cell invasion by down-regulating vimentin expression^32^. In present study, we showed that FAM84B knockdown resulted in tumor growth delay. Taken together, these findings suggest that FAM84B may be involved in cancer initiation and progression and is a potential target for cancer prevention and therapy.

We acknowledge that the present analysis has limitations and weaknesses. The sample size remains relatively small and the results are not robust, demanding further validation in a large and independent cohort. Patients underwent different chemotherapy regimens instead of uniform treatment protocol. Patients were clinically staged according to the American Joint Committee on Cancer 6th edition instead of the latest 7th edition. The exact source of circulating mRNA or serum protein remains a matter of debate. However, to our knowledge, the present analysis is the only one to identify this novel, clinically relevant, circulating biomarker with biological significance using high-throughput technology. Our finding is a preliminary step toward identification of more reliable predictors of response to CRT in clinical practice and potential therapeutic targets in ESCC.

## Methods

### Patients

Between June 2007 and October 2009, patients with biopsy-proven, operable ESCC undergoing neoadjuvant CRT at our institute were prospectively invited to participate and provided informed consent for sample and data collection. All experimental protocols were approved by Institutional Review Board (IRB) of National Taiwan University Hospital. A total of 37 patients who completed the multimodality treatment and provided blood samples before and after neoadjuvant CRT were enrolled. Patients who did not receive radical esophagectomy or provide post-CRT blood samples were excluded. Stage was determined according to the 6th edition of the American Joint Committee on Cancer TNM classification.

### Neoadjuvant treatment

All patients underwent combined modality therapy in accordance to approved procedures. Patients received conformal radiotherapy with 40 Gy in 20 daily fractions, 5 fractions per week over 4 weeks and were treated with concurrent cisplatin-based chemotherapy. The details of chemotherapy regimens are shown in Table 1. Curative surgery with radical esophagectomy and en-bloc lymph node dissection was performed 2–10 weeks (median 7 weeks) after completion of CRT.

### Definition of pathological response

Pathological complete response is defined as no residual tumor is the esophagus or dissected lymph nodes. Presence of any residual esophageal tumor regardless of the percentage of regression, any metastatic lymph node(s), or metastatic disease is considered non-pCR.

### Blood sampling

Whole blood was withdrawn in accordance to an IRB-approved procedure within one week before the start of CRT and within 4 weeks after the completion of CRT.

### Microarray analysis

Total RNA was prepared using the PerfectPure RNA blood kit (5 Prime Inc., Gaithersburg, MD) according to the protocol provided by the manufacturer of Tempus™ tubes. Globin mRNA was depleted from each total RNA sample using the GLOBINclear™-Human kit (Ambion, Austin, TX). RNA concentration and quality were determined using a NanoDrop ND-1000 spectrophotometer (NanoDrop Technologies, Wilmington, DE) and Agilent 2100 Bioanalyzer/RNA 600 LabChip kit (Agilent Technologies, Palo Alto, CA). RNA of sufficient quality with an RNA integrity number >7.0 was used to synthesize cRNA for microarray analysis.

Globin depleted RNA (500 ng) was primed with the T7 Oligo(dT) primer and amplified using an Illumina TotalPrep RNA Amplification kit (Ambion) to synthesize the first strand cDNA containing a T7 promoter sequence. The cDNA then underwent second strand synthesis, RNA degradation by DNA Polymerase and RNase H, and a clean-up process to remove excess RNA, primers, enzymes, and salts that would inhibit *in vitro* transcription. *In vitro* transcription was employed using the double-stranded cDNA as a template and T7 RNA polymerase to synthesize multiple copies of biotinylated-cRNA. The labeled cRNA was purified by a Filter Cartridge and quantified by a NanoDrop spectrophotometer (NanoDrop Technologies, Wilmington, DE). Labeled cRNA (1.5 μg) was hybridized to Illumina Human WG-6 v3 BeadChips (Illumina, San Diego, CA). The intensity of the bead’s fluorescence was detected by the Illumina BeadArray reader and the data analyzed using Bead Studio software. The data were pre-processed using the lumi R/BioConductor package. Array background adjustment was performed using a quantile normalization algorithm, and the data were logarithm base-2 transformed for further statistical analysis.

Probes were filtered out if their detection call *p*-value was >0.01 in all samples (eliminating 31,757 [65%] of 48,804 probes). All data were minimum information about a microarray experiment (MIAME) compliant, and the raw data have been submitted to the Gene Expression Omnibus database (accession number GSE43519).

### Quantitative reverse transcription polymerase chain reaction (qRT-PCR)

Reverse transcription of RNA was performed with a High Capacity cDNA RT kit (Applied Biosystems, Foster City, CA) using random primers and 1 μg of total RNA from samples as template. The Taqman® gene expression assays are used to validate selected candidate genes. Taqman® quantitative real time PCR was carried out using an ABI 7900HT Fast Real-time PCR System (Applied Biosystems, Foster City, CA). The PCR conditions were as follows: 50 cycles of denaturation at 95 °C for 15 sec and 1 min of annealing and elongation at 60 °C. Beta-actin (Hs99999903_m1) was selected as an internal control. All reactions for each target gene and endogenous control were measured in triplicate. Relative gene expression data was calculated using the ∆∆-cycle threshold (CT) method.

### Proximity ligation assay

Multiplex proximal ligation assay (PLA) was performed on serum samples as described previously^15^,^33^. Briefly, samples were thawed and mixed in a 1:1 ratio with buffer (Olink AB, Uppsala, Sweden) for undiluted assay. For probing, 2 μL of the buffered serum sample was mixed with 2 μL of probe, and incubated for 2 hours at 37 °C to allow the probe to bind analytes. Ligation was achieved by incubating the reaction mixture with the probed samples for 15 minutes at 30 °C to dilute and separate any free probes. To stop ligation, 2 μL of uracil-DNA excision mix (Epicentre, Madison, WI) was added and incubated for 15 minutes at room temperature. Preamplification of the bar-coded amplicons was performed with pool-PCR primer (Platinum Taq kit, Invitrogen Corp., Carlsbad, CA) for 13 cycles with a 4-minute extension at 60 °C, and the preamplification products were diluted 10-fold in 1 × TE. For each protein assayed, a separate quantitative PCR reaction was required in a 384-well plate with target protein–specific quantitative detection primer lyophilized at the bottom of each well, to which sample and iTaq SYBR Green Supermix with Rox (Bio-Rad, Hercules, CA) were added. Real-time quantitative PCR was performed with a sample volume of 10 μL per well for 40 cycles at 95 °C for 15 seconds and 60 °C for 1 minute. To ensure standardization of values for each biomarker investigated, all samples were simultaneously probed and evaluated on a single 384-well plate with a PBS-BSA blank well. Cycle threshold (CT) values from quantitative PCR were converted into an estimated number of starting amplicons, or PLA units, by calculating 10 (-0.301 × CT + 11.439).

### Immunohistochemistry analysis

Four-μm sections were cut from the paraffin-embedded tissue samples. Antigen retrieval was performed using Trilogy™ (Cell Marque, Rocklin, CA) at 121 °C for 10 minutes. The sections were treated with 3% hydrogen peroxidase to quench endogenous hydrogen peroxidase activity, treated with Power Block (BioGenex, San Ramon, CA) for 20 minutes to block nonspecific reactions to other antigens, incubated with the commercial FAM84B rabbit polyclonal antibody (1:100; Proteintech Group, Chicago, IL) overnight at 4 °C, stained with diaminobenzidine (DAB) (BioGenex), washed additional times, and counter-stained with hematoxylin for 3 minutes. The staining intensity was scored from 0 to 3+ as follows: 0, no staining; 1+, weak staining; 2+, moderate staining; 3+, strong staining. High intensity was defined as staining intensity of 2 ~ 3+ and 3+, and low intensity was defined as staining intensity from 0 to 2+.

### Cell culture and western blotting

The human non-neoplastic esophageal epithelial cell line Het-1A (American Type Culture Collection [ATCC], Rockville, MD) was maintained in RPMI 1640 (Gibco BRL, Paisley, UK). The human esophageal squamous cell lines CE-48T/VGH, CE-81T/VGH, and CE-146/VGH (Bioresource Collection and Research Center [BCRC], Hsiuchu City, Taiwan) were maintained in DMEM (Gibco), while KYSE-30, KYSE-70, KYSE-150, KYSE-270, KYSE-410, OE-21 were gifts of Dr. Chih-Hung Hsu and cultured in RPMI 1640. All culture media were supplemented with 10% (v/v) fetal bovine serum and 1% antibiotics (penicillin-streptomycin-amphotericin B). Cells were cultured at 37 °C in a humidified atmosphere of 5% CO2/95% air. Mycoplasma contamination was tested on a regular basis. Whole cell lysates were prepared using cell lysis buffer (Cell Signaling, Beverly, MA) plus protease inhibitor cocktail (Thermo Fisher Scientific, Waltham, MA). Protein concentration was determined with Bradford reagent (Bio-Rad, Hercules, CA). Proteins in samples (each containing 100 μg of total protein) were separated by 10% sodium dodecyl sulfate (SDS)-polyacrylamide gel electrophoresis (PAGE), transferred to polyvinylidenedifluoride membranes, and immunoblotted with antibodies against human FAM84B (Abcam, Cambridge, MA) and β-actin (Santa Cruz Biotechnology, Santa Cruz, CA). Bound antibodies were detected using the appropriate horseradish peroxidase-coupled secondary antibodies followed by enhanced chemiluminescence with luminol substrate (Millipore, Bedford, MA).

### Gene expression knockdown

To establish a stable clonal cell population with knockout FAM84B, specific shRNA constructs cloned into plasmid pLKO.1-puro are obtained from The RNAi Consortium (TRC) via National RNAi Core Facility (Academia Sinica, Taiwan ROC). The shRNA-encoding plasmid delivery is conducted by a 3-plasmid lentiviral vector system. Lentiviruses are prepared according to the standard protocol. Cells are infected with FAM84B-targeting pseudovirus in the presence of 8 μg ml^–1^ polybrene followed by 0.5-1.0 μg ml^–1^ puromycin selection at 24 hours after transduction. Knockdown efficiency of the target cells is validated by Western blot. The target sequence used for FAM84B knockdown is “CACCTAAGTTACAAGGAAGTT”.

### *In vivo* ectopic xenograft model

Male SCID mice (6 weeks of age) were obtained from the National Laboratory Animal Center and used for ectopic (subcutaneous) xenograft implantation. Ectopic tumors were established by subcutaneous injection 1 × 10^6^ of wide type CE81T/VGH cells, CE81T/VGH cells with control shRNA, and CE-81T/VGH cells with FAM84B shRNA into the right hind leg of mice. There were 5 mice in each group. Tumor volumes were measured with a set of calipers and calculated using a standard formula: width × length × depth/2. All experimental procedures using these mice were performed in accordance with protocols approved by the National Taiwan University Institutional Animal Care and Use Committee.

### Statistical analysis

The Significance Analysis of Microarrays algorithm with control of false discovery rate (FDR) was used to select the genes that were the most differentially expressed after neoadjuvant CRT. DAVID (Database for Annotation, Visualization, and Integrated Discovery) was used assessed the significance of functional annotation clustering for genes differentially expressed after CRT. To compare pCR versus non-pCR, where the difference between samples was less marked, we used BAMarray 3.0, which performs Bayesian ANOVA on microarray data (BAM) to provide an optimal balance between type I and type II errors. The Wilcoxon Mann-Whitney test and logistic regression analysis were used to evaluate the association between individual gene expression and pathological response, with p value less than 0.01 being considered significant. The predictive performance was evaluated by receiver operating characteristic (ROC) curve analysis. The Chi-square and Fisher’s exact tests were used to assess the relationship of patient characteristics with treatment response. The Kolmogorov-Smirnov test was used to compare the distributions of staining intensity between response groups. The Kaplan-Meier method was used to calculate survival rate and the Log-rank test was used to determine significant differences in survival across the factor. The statistical analyses were performed with SPSS version 19.0 (SPSS Inc., Chicago, IL), and statistical significance was determined with a cutoff p-value less than 0.05. In addition, the ROUT method (implemented in Graphpad Prism version 6.0 [Graphpad Inc., San Diego, CA]) with FDR ≤5% was used to identify outliers from nonlinear regression.

## Author Contributions

H.F.M collected, analyzed and interpreted data, did statistical analysis, and wrote the main manuscript. C. J.C.H originated idea, designed the study, interpreted data, and wrote the main manuscript. C.Y.L supported materials, prepared Table 1 and Fig. 4a. L.J.M supported materials, designed the study, and collected data. K.A.C supported materials, analyze data, and prepared Fig. 3. C.E.Y originate idea, designed the study, interpreted and analyzed data, and write the main manuscript. All authors reviewed the manuscript.

## Additional Information

**How to cite this article**: Hsu, F.-M. *et al.* Circulating mRNA Profiling in Esophageal Squamous Cell Carcinoma Identifies FAM84B As A Biomarker In Predicting Pathological Response to Neoadjuvant Chemoradiation. *Sci. Rep.* **5**, 10291; doi: 10.1038/srep10291 (2015).

## Supplementary Material

Supplementary Table e1

## Figures and Tables

**Table 1:** Patient Characteristics.

**ID**	**Age**	**Gender**	**Clinical** **TNM Stage**	**Induction C/T**	**Concurrent C/T**	**Pathologic Response**	**Pre-TMT FAM84B IHC Intensity**	**Post-TMT FAM84B IHC Intensity**
Microarray & RT-PCR								
316	48	M	T3N1M1b	None	DP	CR	2+	NA
833	62	M	T2N1M0	None	TP	CR	2+	NA
743	42	M	T3N1M1a	None	TP	nCR	NA	NE
040	52	M	T3N1M0	None	DP	nCR	NA	2+
843	45	M	T3N1M1b	TP-HDFL	TP	nCR	3+	3+
146	63	M	T3N0M1a	TP-HDFL	TP	CR	2+	NA
690	55	M	T3N1M0	None	DP	CR	2~3+	NA
817	48	M	T1N1M1a	TP-HDFL	TP	CR	2~3+	NA
153	47	M	T3N1M0	TP-HDFL	TP	CR	NA	NA
694	54	M	T3N1M0	None	PF	nCR	2+	3+
995	53	M	T3N1M0	TP-HDFL	TP	CR	1+	NA
450	49	M	T3N1M1a	TP-HDFL	TP	nCR	3+	2~3+
970	45	M	T3N1M0	None	TP	CR	0	NA
554	64	M	T3N1M0	TP-HDFL	TP	CR	NA	NA
809	52	M	T3N1M1a	None	PF	nCR	2+	2+
490	65	M	T4N1M1b	None	PF	nCR	3+	3+
618	56	M	T3N1M0	TP-HDFL	TP	nCR	1~2+	3+
193	51	M	T3N1M0	None	TP	CR	2~3+	NA
967	62	M	T3N1M0	TP-HDFL	TP	nCR	2+	2+
825	70	M	T3N1M0	TP-HDFL	TP	nCR	2~3+	2+
210	64	M	T3N1M0	None	PF	nCR	2+	2+
RT-PCR only								
067	50	M	T3N1M1b	TP-HDFL	PF	nCR	2+	2+
906	74	M	T3N1M0	None	TP	CR	NA	NA
609	50	M	T3N1M0	TP-HDFL	TP	nCR	2+	NE
887	48	M	T3N1M0	None	DP	nCR	2+	3+
951	55	F	T3N1M0	None	TP	nCR	2+	1~2+
486	50	M	T3N0M0	TP-HDFL	TP	CR	3+	NA
557	70	M	T3N1M0	None	TP	nCR	3+	2~3+
085	37	M	T3N1M0	None	DP	nCR	2~3+	NE
834	50	M	T3N1M0	None	CTP	nCR	0~1+	2+
181	49	M	T3N1M0	None	CTP	CR	2+	NA
890	63	M	T3N1M0	None	CTP	CR	2+	NA
532	37	M	T3N0M0	None	CTP	nCR	2~3+	NE
141	64	M	T3N1M0	None	TP	CR	1~2+	NA
847	40	M	T3N1M0	None	CTP	nCR	1+	2+
632	47	M	T3N1M0	None	CTP	CR	0	NA
565	47	M	T3N1M0	None	CTP	nCR	1+	2+

Abbreviations: C/T, chemotherapy; TMT, tri-modality therapy, IHC, immunohistochemistry; M, male; F, female; CR, complete response; nCR, non-complete response; NA, not available; NE: not evaluable.

Chemotherapy regimens: TP-HDFL, paclitaxel (80 mg/m^2^) on days 1 and 8, cisplatin (35 mg/m^2^) on days 2 and 9, fluorouracil (2000 mg/m^2^) and leucovorin (300 mg/m^2^) on days 2 and 9; PF, cisplatin (30 mg/m^2^) and fluorouracil (425 mg/m^2^) once weekly; TP, paclitaxel (35 mg/m^2^) on days 1 and 4 of each week and cisplatin (15 mg/m^2^) on days 2 and 5 of each week; DP, docetaxel (20 mg/m^2^) and cisplatin (20 mg/m^2^) once weekly; CTP, cetuximab (400 mg/m^2^) on week -1 and (250 mg/m^2^) once weekly, paclitaxel (35 mg/m^2^) on days 1 and 4 of each week and cisplatin (15 mg/m^2^) on days 2 and 5 of each week.

**Table 2:** List of Altered mRNAs Differentially Expressed Between pCR and non-pCR.

**mRNA Symbol**	**Absolute BAM Zcut Value**	**Regulation**	***P*** **Value of Logistic Regression**	***P*** **Value of Wilcoxon Mann-Whitney Test**
AFTPH	3.15	Down	0.01*	0.009*
C10ORF76	2.51	Down	0.018	0.024
CCNL1	3.41	Down	0.005**	0.004**
FAM13A1	3.62	Up	0.003**	0.005*
FAM84B	4.13	Down	0.003**	0.001**
HIST1H4H	3.69	Up	0.01	0.017
HIST2H4A	3.20	Up	0.007*	0.009*
IFI27	4.45	Down	0.08	0.105
KCNRG	3.39	Up	0.008*	0.014
SEPT4	3.85	Up	0.002**	0.003**

Abbreviations: BAM, Bayesian ANOVA for microarray; pCR, pathological complete response; * indicates *p* value ≤0.01; ** indicated *p* value ≤0.005.
