# Supplementary material for: Circulating mRNA Profiling in Esophageal Squamous Cell Carcinoma Identifies FAM84B As A Biomarker In Predicting Pathological Response to Neoadjuvant Chemoradiation
Source: Sci Rep. 2015 May 18;5:10291. doi: 10.1038/srep10291 (PMC4434848; doi:10.1038/srep10291)
Supplement: Supplementary Table e1 [file srep10291-s1.pdf]

Manuscript Title

Circulating mRNA Profiling in Esophageal Squamous Cell Carcinoma Identifies FAM84B As A Biomaker In Predicting Pathological Response to Neoadjuvant Chemoradiation

Authors: Feng-Ming Hsu, Jason Chia-Hsien Cheng, Yih-Leong Chang, Jang-Ming Lee, Albert C. Koong, Eric Y. Chuang

Supplementary Table e1. David (Database for Annotation, Visualization, and Integrated Discovery) functional annotation clustering with differentially expressed genes before and after chemoradiation.

Annotation Cluster 1	Enrichment Score: 8.64			
Category	Term	Count	P_Value	Bonferroni
GOTERM_BP_ALL	GO:0002376~immune system process	33	1.2E-14	1.3E-11
GOTERM_BP_ALL	GO:0006955~immune response	24	7.2E-11	7.7E-8
GOTERM_BP_ALL	GO:0050896~response to stimulus	34	1.4E-2	1.0E0
Annotation Cluster 2	Enrichment Score: 2.84			
Category	Term	Count	P_Value	Bonferroni
GOTERM_BP_ALL	GO:0048534~hemopoietic or lymphoid organ development	10	5.3E-5	5.5E-2
GOTERM_BP_ALL	GO:0002520~immune system development	10	8.4E-5	8.6E-2
GOTERM_BP_ALL	GO:0046649~lymphocyte activation	8	3.4E-4	3.0E-1
GOTERM_BP_ALL	GO:0048872~homeostasis of number of cells	6	5.1E-4	4.2E-1
GOTERM_BP_ALL	GO:0030097~hemopoiesis	8	9.3E-4	6.3E-1
GOTERM_BP_ALL	GO:0045321~leukocyte activation	8	1.1E-3	6.9E-1
GOTERM_BP_ALL	GO:0042113~B cell activation	5	1.6E-3	8.2E-1
GOTERM_BP_ALL	GO:0046651~lymphocyte proliferation	4	2.6E-3	9.4E-1
GOTERM_BP_ALL	GO:0001775~cell activation	8	2.9E-3	9.5E-1
GOTERM_BP_ALL	GO:0070661~leukocyte proliferation	4	3.0E-3	9.6E-1

GOTERM_BP_ALL	GO:0032943~mononuclear cell proliferation	4	3.0E-3	9.6E-1
GOTERM_BP_ALL	GO:0042100~B cell proliferation	3	3.2E-3	9.7E-1
GOTERM_BP_ALL	GO:0048538~thymus development	3	9.1E-3	1.0E0
GOTERM_BP_ALL	GO:0042110~T cell activation	5	9.6E-3	1.0E0
GOTERM_BP_ALL	GO:0002521~leukocyte differentiation	5	1.1E-2	1.0E0
Annotation Cluster 3	Enrichment Score: 2.61			
Category	Term	Count	P_Value	Bonferroni
GOTERM_BP_ALL	GO:0002682~regulation of immune system process	11	2.2E-4	2.1E-1
GOTERM_BP_ALL	GO:0050776~regulation of immune response	8	7.2E-4	5.4E-1
GOTERM_BP_ALL	GO:0002684~positive regulation of immune system process	8	9.8E-4	6.5E-1
GOTERM_BP_ALL	GO:0002768~immune response-regulating cell surface receptor signaling pathway	4	2.6E-3	9.4E-1
GOTERM_BP_ALL	GO:0050778~positive regulation of immune response	6	2.7E-3	9.4E-1
GOTERM_BP_ALL	GO:0002253~activation of immune response	5	3.4E-3	9.7E-1
GOTERM_BP_ALL	GO:0048583~regulation of response to stimulus	10	3.5E-3	9.8E-1
GOTERM_BP_ALL	GO:0048584~positive regulation of response to stimulus	7	4.7E-3	9.9E-1
GOTERM_BP_ALL	GO:0002764~immune response-regulating signal transduction	4	6.0E-3	1.0E0
GOTERM_BP_ALL	GO:0050851~antigen receptor-mediated signaling pathway	3	2.0E-2	1.0E0
Annotation Cluster 4	Enrichment Score: 2.09			
Category	Term	Count	P_Value	Bonferroni
GOTERM_BP_ALL	GO:0042440~pigment metabolic process	4	3.2E-3	9.7E-1
GOTERM_BP_ALL	GO:0006783~heme biosynthetic process	3	5.5E-3	1.0E0
GOTERM_BP_ALL	GO:0033014~tetrapyrrole biosynthetic process	3	7.6E-3	1.0E0
GOTERM_BP_ALL	GO:0006779~porphyrin biosynthetic process	3	7.6E-3	1.0E0
GOTERM_BP_ALL	GO:0042168~heme metabolic process	3	9.1E-3	1.0E0
GOTERM_BP_ALL	GO:0033013~tetrapyrrole metabolic process	3	1.6E-2	1.0E0
GOTERM_BP_ALL	GO:0006778~porphyrin metabolic process	3	1.6E-2	1.0E0
Annotation Cluster 5	Enrichment Score: 1.96			
Category	Term	Count	P_Value	Bonferroni
GOTERM_BP_	GO:0006959~humoral immune response	5	1.8E-3	8.6E-1

ALL				
GOTERM_BP_ ALL	GO:0006952~defense response	11	7.2E-3	1.0E0
GOTERM_BP_ ALL	GO:0042592~homeostatic process	12	1.0E-2	1.0E0
GOTERM_BP_ ALL	GO:0019725~cellular homeostasis	9	1.2E-2	1.0E0
GOTERM_BP_ ALL	GO:0030005~cellular di-, tri-valent inorganic cation homeostasis	6	1.7E-2	1.0E0
GOTERM_BP_ ALL	GO:0006879~cellular iron ion homeostasis	3	1.8E-2	1.0E0
GOTERM_BP_ ALL	GO:0055066~di-, tri-valent inorganic cation homeostasis	6	2.1E-2	1.0E0
GOTERM_BP_ ALL	GO:0055072~iron ion homeostasis	3	2.3E-2	1.0E0
